# Applications of Computer-Aided Design and Computer-Aided Manufacturing in Pediatric Orthodontics: A Scoping Review

**DOI:** 10.7759/cureus.112715

**Published:** 2026-07-15

**Authors:** Bianca Campos de Freitas, Ana Carla do Espírito Santo Rodrigues Melo, Vinícius Rodrigues Reis de Farias, Matheus Boaventura Malaquias Reis, Marcelo da Luz Silva Lima, Fernanda Guzzo Tonial

**Affiliations:** 1 Pediatric Dentistry, Afya Faculdade de Ciências Médicas, Palmas, BRA

**Keywords:** computer-aided design and computer-aided manufacturing (cad/cam), digital orthodontics, interceptive orthodontics, orthodontic appliances, pediatric and preventive dentistry

## Abstract

Computer-aided design/computer-aided manufacturing (CAD/CAM) technology has been increasingly incorporated into dentistry, expanding the possibilities of digital workflows through greater precision, customization, and optimization of clinical procedures. In pediatric orthodontics, CAD/CAM has been applied in different clinical contexts, although its use is still evolving. This scoping review aimed to identify and synthesize the applications of CAD/CAM in preventive and interceptive orthodontics in children, considering the devices used and the main reported outcomes. The search was conducted in the National Library of Medicine (PubMed), Scientific Electronic Library Online (SciELO), Latin American and Caribbean Health Sciences Literature (LILACS), and Google Scholar databases up to April 2026, with no restriction on publication year. Ultimately, 18 studies were considered eligible, including randomized clinical trials, prospective and retrospective clinical studies, case series, and case reports. The main applications involved the fabrication of space maintainers, functional devices, fixed orthodontic prostheses, interceptive removable appliances, and nasoalveolar molding in neonates with cleft lip and palate. The included studies demonstrated heterogeneous clinical outcomes, particularly regarding the survival of digital devices compared with conventional methods. Overall, the available evidence suggests that CAD/CAM technology may provide advantages related to device customization, adequate clinical adaptation, reduced clinical time, and optimization of clinical management in selected clinical contexts. However, comparative studies reported inconsistent survival outcomes, precluding definitive conclusions regarding the comparative clinical performance of CAD/CAM and conventional approaches. The available evidence remains limited, with a predominance of descriptive studies, highlighting the need for well-designed prospective studies with greater methodological rigor and long-term follow-up to better define the clinical impact of CAD/CAM applications in pediatric orthodontics.

## Introduction and background

The incorporation of digital technologies has promoted important innovations in dentistry, with a direct impact on diagnostic accuracy and treatment predictability [1]. In this context, the computer-aided design/computer-aided manufacturing (CAD/CAM) workflow has become established as one of the main innovations, enabling the digitization, planning, and fabrication of customized devices through integrated systems. Continuous advances have made these technologies increasingly accessible, with more user-friendly interfaces and a broader range of applications [2,3].

In pediatric dentistry, CAD/CAM has been incorporated into clinical practice as an alternative to conventional techniques, particularly in situations where conventional clinical and laboratory workflows may be challenging in children. A systematic review with meta-analysis involving 337 children demonstrated that digital intraoral scanning, compared with conventional impressions, was associated with shorter procedure time, greater patient comfort, reduced pain, and a lower gag reflex, supporting its use as a favorable alternative to conventional impression techniques [4]. Although these findings are encouraging, the successful implementation of digital workflows in pediatric patients should also consider patient-related and clinical factors that may influence image acquisition and treatment execution.

Early orthodontic intervention plays an important role in preserving oral function and guiding craniofacial development in children, particularly in the presence of developing malocclusions, which are highly prevalent and negatively impact quality of life, constituting an important public health concern [5,6]. In this setting, CAD/CAM has been associated with improved patient cooperation and reduced clinical time [1,7]. In addition, CAD/CAM enables the fabrication of customized space maintainers, functional devices, and removable appliances, favoring clinical adaptation and treatment outcomes [8]. CAD/CAM has also been applied in clinical situations requiring more individualized approaches, including early tooth loss, craniofacial malformations, and patients with limited cooperation [7].

Despite the advances in CAD/CAM in orthodontics, the available reviews have predominantly focused on corrective treatments. Existing studies have evaluated the application of CAD/CAM in customized brackets, retainers, and indirect bonding, with limited attention to preventive and interceptive orthodontic interventions [9-11]. Given the specific characteristics of pediatric orthodontics, including primary and mixed dentition, active craniofacial growth, and the need for individualized appliances, it is important to map the available evidence regarding the application of CAD/CAM technologies in early orthodontic and craniofacial interventions in children. Therefore, this scoping review aimed to map and synthesize the available evidence regarding the applications of CAD/CAM technology in pediatric orthodontics.

## Review

Methods

Study Design

The present study was conducted as a scoping review, in accordance with the methodological recommendations of the Joanna Briggs Institute [12], and reported according to the Preferred Reporting Items for Systematic Reviews and Meta-Analyses extension for Scoping Reviews (PRISMA-ScR) checklist [13]. The protocol was registered in the Open Science Framework (OSF) on April 20, 2026, and received the following DOI: 10.17605/OSF.IO/F72TP.

Research Question

According to the criteria established for scoping reviews, the Population, Concept, and Context (PCC) framework was used to formulate the guiding question. The components were defined as follows: Population (P): children in the primary and/or mixed dentition requiring orthodontic intervention; Concept (C): applications of CAD/CAM technology, including digital workflow in the planning, design, and fabrication of orthodontic devices; Context (C): preventive and/or interceptive orthodontics. Based on these elements, the following question was addressed: What are the applications of CAD/CAM technology in preventive and interceptive orthodontics in children, and how are they characterized in terms of devices and clinical indications?

Eligibility Criteria

Primary studies with direct clinical application of CAD/CAM in preventive and interceptive orthodontics were included, comprising randomized clinical trials, prospective and retrospective clinical studies, case series, and case reports. The selection included patients in the primary or mixed dentition, regardless of sex, systemic condition, or treatment setting. In contrast, studies involving patients in permanent dentition (adolescents and adults), laboratory studies (in vitro), technical reports, letters to the editor, and secondary studies were excluded.

Search Strategy

The electronic search was conducted in the National Library of Medicine (PubMed), Latin American and Caribbean Health Sciences Literature (LILACS), and Scientific Electronic Library Online (SciELO) using controlled vocabulary (MeSH/DeCS) and free-text terms. Search strategies were adapted to the indexing characteristics of each database. While the PubMed search included more specific terms related to CAD/CAM applications and orthodontic appliances, the LILACS and SciELO searches used broader descriptors to accommodate the indexing characteristics of these regional databases. A complementary search was also performed in Google Scholar to identify potentially relevant studies not retrieved through the primary databases. Considering the broad indexing and limited reproducibility of Google Scholar, a simplified search strategy was adopted for this complementary search. The complete search strategies used for study identification are presented in Table 1.

**Table 1:** Search strategies used for study identification

	Search strategy
PubMed	(("Computer-Aided Design"[MeSH] OR "Computer-Aided Manufacturing"[MeSH] OR CAD/CAM OR "digital workflow" OR "intraoral scan*" OR "intraoral scanner*"OR "3D print*") AND ("Orthodontics"[MeSH] OR orthodontic* OR "interceptive orthodontics" OR "preventive orthodontics" OR "space maintainer*" OR "palatal expan*" OR hyrax OR haas OR distalizer OR "functional appliance*" OR "clear aligners") AND ("Child"[MeSH] OR child* OR pediatric* OR paediatric* OR "mixed dentition" OR "primary dentition"))
LILACS	tw:("CAD/CAM" OR "Computer-Aided Design" OR "Computer-Aided Manufacturing" OR "Desenho Assistido por Computador" OR "Fabricação Assistida por Computador" OR "fluxo digital" OR "escaneamento intraoral") AND tw:("Odontopediatria" OR "Odontologia Pediátrica" OR "Pediatric Dentistry" OR "Ortodontia Interceptora" OR "Orthodontics, Interceptive" OR "Ortodontia Preventiva" OR "Orthodontics, Preventive" OR "Ortodontia" OR "Orthodontics")
SciELO	("CAD/CAM" OR "Computer-Aided Design" OR "Desenho Assistido por Computador") AND ("Odontopediatria" OR "Odontologia Pediátrica" OR "Pediatric Dentistry" OR "Ortodontia Interceptora" OR "Orthodontics, Interceptive" OR "Ortodontia Preventiva" OR "Orthodontics, Preventive" OR "Ortodontia" OR "Orthodontics")
Google Scholar	"digital orthodontics" AND "children"

Data Analysis

The identified references were exported and managed using the Rayyan platform (Rayyan Systems Inc., Cambridge, MA, USA; available at: https://www.rayyan.ai/). The study selection process followed the stages of identification, screening, eligibility, and inclusion. Initially, studies were assessed through the reading of titles and abstracts and, subsequently, through full-text reading of potentially eligible studies. Studies that answered the proposed research question were included.

Study selection was conducted by two independent researchers, and any disagreements regarding eligibility were resolved by consensus, based on individual analysis of the studies. After selection, data were extracted and organized in a Microsoft Excel spreadsheet (Microsoft Corp., Redmond, WA, USA) by the principal researcher, including information on author(s), year of publication, study design, type of CAD/CAM technology application, device, clinical indication, and main findings. The results were synthesized descriptively and presented in tabular and narrative form, according to the objectives and research question of the review.

Results

**Figure 1:**
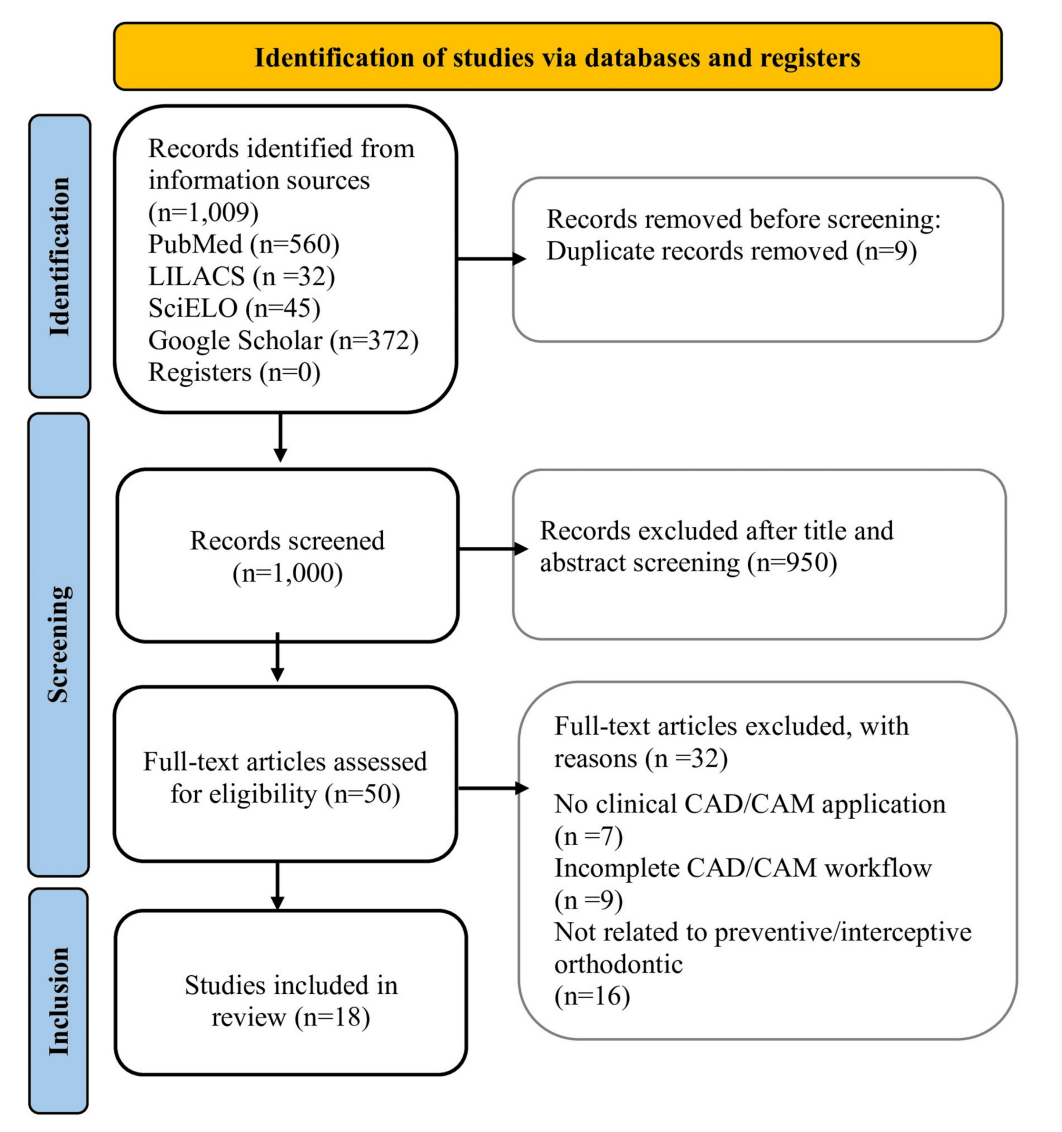
PRISMA-ScR flowchart detailing the article selection process PRISMA-ScR: Preferred Reporting Items for Systematic Reviews and Meta-Analyses extension for Scoping Reviews; CAD/CAM: computer-aided design/computer-aided manufacturing

A total of 1,009 records were identified through database searches and complementary searches. After duplicate removal and screening of titles and abstracts, 50 studies were selected for full-text reading. Of these, studies that did not meet the eligibility criteria were excluded. At the end of the selection process, 18 studies were included in the review. The study selection process is presented in the PRISMA-ScR flowchart (Figure 1).

The included studies presented different methodological designs, including randomized clinical trials, prospective and retrospective clinical studies, case series, and case reports. The findings were described according to the characteristics of the studied population and the main clinical applications of CAD/CAM technology in pediatric orthodontics.

Population Characteristics

The population consisted of pediatric patients, including neonates and children in the primary and mixed dentition stages, with ages ranging from birth to approximately 12 years. Some studies included patients with specific behavioral management needs, such as attention-deficit/hyperactivity disorder (ADHD) and autism spectrum disorder (ASD) [14,15]. The main clinical condition observed was early loss of primary teeth, especially molars [14-27]. Patients with early loss of maxillary incisors [28], children with anterior crossbite [29], and neonates with cleft lip and palate [30,31] were also included.

Applications of CAD/CAM in Pediatric Orthodontics

The main application of CAD/CAM was the fabrication of space-maintaining devices following early loss of primary teeth, with predominance of band-and-loop maintainers [14,15,18,19,21-23,25]. The digital workflow involved intraoral scanning, planning using CAD software, and fabrication through three-dimensional (3D) printing or milling.

Functional devices and polyetheretherketone (PEEK)-based devices were used for space maintenance associated with functional rehabilitation, with reports of space preservation and restoration of masticatory function during the follow-up period [17,20,24,26,28]. Other devices included digital lingual arches, zirconia-based maintainers, and a transparent removable appliance for anterior crossbite correction, demonstrating adequate clinical adaptation and structural integrity during the evaluated periods [16,27,29].

In addition, CAD/CAM technology was applied in neonates with cleft lip and palate through digital nasoalveolar molding (NAM), involving the planning and fabrication of individualized devices. The studies reported a reduction in cleft dimensions throughout follow-up, including an approximate mean reduction of 5.38 mm in the anterior region and 3.39 mm in the posterior region [30]. The use of digital NAM with double aligners (2A-NAM) was also described, showing adequate device adaptation and control of maxillary segment positioning during treatment [31].

The characteristics of the included studies, including study design, clinical application, type of device, clinical indication, and main findings related to the use of CAD/CAM technology, are presented in Table 2.

**Table 2:** Characteristics of the studies included in the review 3D: three-dimensional; CAD/CAM: computer-aided design/computer-aided manufacturing; NAM: nasoalveolar molding; PEEK: polyetheretherketone; ADHD: attention deficit hyperactivity disorder; ASD: autism spectrum disorder; 2A-NAM: double aligner nasoalveolar molding

Author/year	Study design	Application	Device	Clinical indication	Main findings
Thakur et al. (2024) [22]	Randomized clinical trial (n = 62)	Preventive	3D laser-sintered band-and-loop space maintainer vs. conventional	Early loss of primary molars	Survival: 77.4% (3D) vs. 51.6% (conventional) at 9 months. Gingival health: mild inflammation in both groups during follow-up. Patient satisfaction: >90% in both groups.
Nedeljkovic et al. (2025) [24]	Randomized clinical trial (n = 28)	Preventive	CAD/CAM functional maintainer 3D printing (metal) vs. milling (composite)	Early loss of primary first molar	Clinical performance: 87% device retention and 0% fractures (3D metal) vs. 100% fractures (milled composite) at the 6-month follow-up. Masticatory performance: improved in both groups. Bite force: no significant difference between groups.
Kapoor et al. (2025) [23]	Randomized clinical trial (n = 40)	Preventive	3D band-and-loop space maintainer vs. conventional vs. prefabricated	Early loss of primary molars	Survival: 85% (conventional), 65% (3D), and 30% (prefabricated) at 9 months. Gingival health: no significant difference among groups. Patient satisfaction: similar between conventional and 3D devices, both higher than prefabricated maintainers.
Cengiz and Karayilmaz (2024) [21]	Prospective clinical study (n = 65)	Preventive	3D laser-sintered band-and-loop space maintainer vs. conventional	Early loss of primary molars	Failure rate: 66% (3D) and 38% (conventional). Main cause of failure: decementation. Patient experience: digital impressions were preferred over conventional impressions.
Frazao et al. (2025) [30]	Retrospective clinical study (n = 25)	Early interceptive	NAM with transparent aligners	Complete unilateral cleft lip and palate	Anterior cleft width: reduced by 5.38 mm. Posterior cleft width: reduced by 3.39 mm.
Wang et al. (2023) [20]	Case series (n = 15)	Preventive	Semi-rigid PEEK bridge	Early loss of primary molar	Space maintenance: no significant space loss during the 6-month follow-up. Clinical performance: all devices remained stable, without dislodgement or fracture. Masticatory function: restored.
Umamaheswari et al. (2025) [25]	Case series (n = 10)	Preventive	3D DMLS (direct metal laser sintering) band-and-loop space maintainer	Early loss of primary molars	Survival: significantly improved during the 6-month follow-up. Gingival health: gingival index significantly increased over time. Patient satisfaction: high.
Ierardo et al. (2017) [17]	Case series (n = 3)	Preventive	PEEK space maintainers, lingual arch, band-and-loop, removable plate	Early loss and need for space maintenance	Space maintenance: maintained during the 9-month follow-up. Clinical performance: no decementation or fractures. Patient acceptance: good.
Zhang et al. (2021) [29]	Case series (n = 2)	Interceptive	Transparent removable appliance with 3D posterior bite plane	Anterior crossbite in primary dentition	Anterior crossbite correction: achieved within 2-6 months, with normal overjet and overbite. Clinical outcome: improved dental and soft tissue relationships. Patient satisfaction: high.
Beretta et al. (2022) [28]	Case series (n = 2)	Preventive	Digital fixed orthodontic prosthesis in PEEK	Early loss of primary incisors	Esthetic-functional rehabilitation: successfully achieved. Space maintenance: maintained during follow-up. Clinical performance: comfortable and easy to apply.
Rathi et al. (2023) [14]	Case series (n = 2)	Preventive	3D metallic band-and-loop space maintainer	Early loss of mandibular primary first molar in children with ADHD	Clinical feasibility: demonstrated in children with ADHD. Clinical performance: successful 6-month follow-up without reported complications.
Soni (2017) [16]	Case report (n = 1)	Preventive	CAD/CAM esthetic zirconia maintainer (modified adhesive band-and-loop type)	Early loss of maxillary primary first molar	Clinical stability: observed at the 6-month follow-up. Gingival health: absence of gingival inflammation. Masticatory function: preserved.
Pawar (2019) [18]	Case report (n = 1)	Preventive	3D band-and-loop space maintainer	Early loss of mandibular primary first molar	Clinical feasibility: demonstrated for 3D-printed space maintainer fabrication. Clinical adaptation: satisfactory.
Khanna et al. (2021) [19]	Case report (n = 1)	Preventive	Conventional vs. 3D band-and-loop space maintainer	Bilateral early loss of primary first molars	Device integrity: the 3D maintainer remained intact at the 6-month follow-up. Conventional device: signs of crack/fracture in the soldering region.
Yangdol et al. (2023) [15]	Case report (n = 1)	Preventive	3D metallic band-and-loop space maintainer	Early loss of mandibular primary second molar in a child with ASD	Clinical feasibility: demonstrated in a child with ASD. Chairside time: reduced.
Trivedi et al. (2024) [27]	Case report (n = 1)	Preventive	3D lingual arch	Multiple early loss of mandibular primary molars	Clinical adaptation: proper band adaptation and fit. Occlusion: no occlusal interferences.
Mladenovic et al. (2025) [26]	Case report (n = 1)	Preventive	3D functional space maintainer	Early loss of primary first molar	Clinical performance: no detachment or breakage during the 12-month follow-up. Masticatory performance: improved.
Rico-Lillo et al. (2026) [31]	Case report (n = 1)	Early interceptive	NAM with double aligners (2A-NAM)	Complete unilateral cleft lip and palate	Alveolar cleft reduction: 5 mm. Digital planning: high agreement between planned and achieved anatomical changes.

Discussion

CAD/CAM technology has been applied in pediatric orthodontics in different clinical contexts, including the fabrication of space maintainers, functional devices, fixed orthodontic prostheses, interceptive removable appliances, and NAM in neonates with cleft lip and palate. Although the included studies demonstrate the diversity of applications, the evidence remains heterogeneous, with a predominance of case reports, small clinical series, and few comparative and randomized studies. This pattern indicates that digital workflows in pediatric patients are promising but still in the process of scientific consolidation.

In comparative studies, the results related to the survival of band-and-loop space maintainers were inconsistent. A survival rate of 65% was observed for 3D devices, lower than that of conventional devices (85%) [23]. Similarly, a higher incidence of failure was identified in 3D devices (66%), mainly due to decementation [21]. In contrast, greater survival of CAD/CAM devices (77.4%) compared with conventional devices (51.6%) after nine months of follow-up was reported [22]. Additionally, greater clinical stability of functional devices produced by metallic 3D printing was demonstrated, with 87% remaining in place after six months, whereas composite-milled devices showed failure in 100% of cases during the same period [24]. Collectively, these findings suggest that the variability in clinical outcomes cannot be attributed solely to the digital workflow itself. The comparative studies differed in manufacturing methods, material characteristics, appliance design, cementation protocols, follow-up periods, evaluated clinical outcomes, and overall study methodology, making direct comparisons difficult and precluding attribution of the observed differences in device survival to a single variable.

Among these comparative studies, the findings reported by Nedeljkovic et al. [24] deserve particular attention. Complete structural failure was observed in the composite-milled functional space maintainers, with fractures occurring in all devices during the six-month follow-up, whereas no fractures were observed in the additively manufactured metal group. Although these findings originate from a single randomized clinical trial and should therefore be interpreted with caution, they suggest that both the manufacturing method and the mechanical properties of the material may substantially influence the structural durability of CAD/CAM appliances subjected to pediatric occlusal loading. These results reinforce the need for further comparative studies before definitive recommendations regarding the most appropriate manufacturing technique or material can be established.

In addition to the clinical outcomes related to device longevity, another relevant aspect was the application of CAD/CAM in contexts requiring greater therapeutic control and behavioral management. In the case of NAM, used in neonates with cleft lip and palate, the results were consistent regarding cleft reduction. A mean reduction of 5.38 mm in the anterior region and 3.39 mm in the posterior region was observed [30], while an approximate 5 mm reduction in alveolar cleft width was also reported, with high predictability between digital planning and clinical outcome [31]. In another context, it was highlighted that the reduction of clinical and laboratory steps in the digital workflow favors behavioral management, expanding the applicability of the technology in patients with ADHD and ASD [14,15]. These findings suggest that CAD/CAM is not limited to the clinical performance of devices but also contributes to optimizing care in more complex pediatric scenarios.

Case reports and small case series consistently demonstrated the feasibility of digital workflows in pediatric orthodontic practice. Greater structural integrity of the 3D space maintainer compared with the conventional device in the same patient was reported [19], as well as adequate clinical adaptation [18]. Furthermore, CAD/CAM functional devices showed satisfactory performance, particularly because they combined space maintenance with functional rehabilitation. Clinical stability over a 12-month period, accompanied by improvement in masticatory function, was observed [26], as well as stability without failures, with space maintenance and restoration of masticatory function using a PEEK device [20], reinforcing that CAD/CAM enables the creation of customized devices capable of efficiently integrating space maintenance and functional rehabilitation. Although these reports demonstrate the feasibility of CAD/CAM applications in pediatric orthodontics, their findings should be interpreted cautiously because they are based on low-level evidence and limited sample sizes.

The diversity of materials used also contributes to the interpretation of the results. PEEK, a thermoplastic polymer with good mechanical resistance and biocompatibility, has been used as a metal-free alternative, with favorable results in terms of adaptation and function [17,20,28]. Zirconia, in turn, stood out as an esthetic material, with adequate tissue response and maintenance of masticatory function during the six-month follow-up [16]. Metallic devices produced by direct metal laser sintering (DMLS) were associated with greater structural integrity and elimination of weld-related failures [15,19,25,27]. Although these findings suggest that material properties and manufacturing methods may influence clinical performance, these variables were not evaluated in isolation, and most available evidence is derived from observational studies. Therefore, no definitive conclusions regarding the superiority of specific materials can be drawn.

This review maps the current applications of CAD/CAM technologies in pediatric orthodontics and highlights their potential to expand therapeutic possibilities across different clinical indications. The available evidence suggests that digital workflows may facilitate the customization of orthodontic devices according to individual patient needs, particularly in more complex clinical scenarios. However, these findings should be interpreted with caution. In accordance with the methodological framework for scoping reviews, a formal critical appraisal of the included studies was not performed, as the primary objective was to map the available evidence rather than assess the effectiveness of specific interventions. Moreover, the predominance of case reports and small case series, together with the heterogeneity of study designs and outcomes, limits the certainty of the available evidence, precluding direct comparisons and definitive conclusions regarding clinical superiority.

Therefore, long-term prospective studies with larger samples and standardized protocols are needed. Future research should prioritize randomized clinical trials comparing CAD/CAM and conventional space maintainers, studies evaluating different manufacturing methods and materials, standardized outcome measures, cost-effectiveness analyses, and patient-reported outcome measures (PROMs), particularly in children requiring behavioral management. Only consistent comparative clinical evidence will make it possible to determine which CAD/CAM applications provide real benefits compared with conventional methods already established in pediatric orthodontics.

## Conclusions

This scoping review mapped the current applications of CAD/CAM technology in pediatric orthodontics across different clinical contexts, including space maintainers, functional appliances, and early interventions related to craniofacial development. The available evidence suggests that CAD/CAM technologies may offer advantages such as device customization, satisfactory clinical adaptation, and optimization of certain aspects of the clinical workflow. However, the current evidence remains limited and heterogeneous, with a predominance of descriptive studies, small sample sizes, and variability in the clinical outcomes assessed, precluding definitive conclusions regarding their clinical superiority over conventional approaches. Therefore, well-designed prospective comparative studies with standardized methodologies, standardized outcome measures, and long-term follow-up are needed to better establish the clinical indications, performance, and long-term outcomes of CAD/CAM applications in pediatric orthodontics.
